# cPath: open source software for collecting, storing, and querying biological pathways

**DOI:** 10.1186/1471-2105-7-497

**Published:** 2006-11-13

**Authors:** Ethan G Cerami, Gary D Bader, Benjamin E Gross, Chris Sander

**Affiliations:** 1 Computational Biology Center, Memorial Sloan-Kettering Cancer Center 1275 York Avenue, Box 460, New York, NY 10021, USA; 2 Banting and Best Department of Medical Research, Terrence Donnelly Centre for Cellular and Biomolecular Research, University of Toronto, 160 College St, Toronto, Ontario M5S 3E1, Canada

## Abstract

**Background:**

Biological pathways, including metabolic pathways, protein interaction networks, signal transduction pathways, and gene regulatory networks, are currently represented in over 220 diverse databases. These data are crucial for the study of specific biological processes, including human diseases. Standard exchange formats for pathway information, such as BioPAX, CellML, SBML and PSI-MI, enable convenient collection of this data for biological research, but mechanisms for common storage and communication are required.

**Results:**

We have developed cPath, an open source database and web application for collecting, storing, and querying biological pathway data. cPath makes it easy to aggregate custom pathway data sets available in standard exchange formats from multiple databases, present pathway data to biologists via a customizable web interface, and export pathway data via a web service to third-party software, such as Cytoscape, for visualization and analysis. cPath is software only, and does not include new pathway information. Key features include: a built-in identifier mapping service for linking identical interactors and linking to external resources; built-in support for PSI-MI and BioPAX standard pathway exchange formats; a web service interface for searching and retrieving pathway data sets; and thorough documentation. The cPath software is freely available under the LGPL open source license for academic and commercial use.

**Conclusion:**

cPath is a robust, scalable, modular, professional-grade software platform for collecting, storing, and querying biological pathways. It can serve as the core data handling component in information systems for pathway visualization, analysis and modeling.

## Background

### Pathway Data Collection for Biology

The complete sequencing of the genomes of numerous organisms provides a genetic "parts list" for human and many model organisms [1,2]. However, a blueprint for how these parts are assembled is required to explain how the system works [3]. This wiring diagram for a cell consists of multiple biological data types, including metabolic pathways, signal transduction pathways, protein-protein interaction networks, gene regulatory networks and genetic interactions. For example, the galactose utilization pathway which allows yeast to harvest energy from galactose sugar consists of a well-studied series of biochemical reactions responsible for converting galactose into glucose-6-phosphate, and a gene regulatory mechanism for switching the pathway on or off [4]. Aberrations within specific pathways have been implicated in many human diseases [5]. For example, the p53 tumor-suppressor pathway has important functions in regulating cell proliferation, and is disrupted in many human cancers [6].

Having interaction and pathway data available in computable form enables the construction of *in silico *models of complex biological processes according to specific hypotheses about how the process works. Such hypotheses can then be verified or falsified by experiment and the models further refined [4]. In the longer term, it may be possible to study the complete "integrated circuit" of a cell, and create mathematical models for understanding which perturbations within the circuit give rise to cancer [5]. Computational modeling of pathways may enable the identification and classification of common logic modules within the cell [7], better enable mechanistic based drug development [3], facilitate the rational design of combination therapies [6], and accelerate the speed of clinical trials [8].

Central to any of these endeavors is the creation and maintenance of interaction and pathway databases. Because of the need to store and organize growing amounts of pathway information and the lack of a common point of organization, the number of interaction databases is growing rapidly, and over 220 exist in the year 2006 [2,9]. Unfortunately, as is the case with many biological data resources [10], interaction and pathway databases have unique data models, distinct access methods, different file formats, and subtle semantic differences [9]. This diversity of implementation makes it extremely difficult to collect data from multiple sources, and therefore slows down scientific research involving pathways [11,12].

To make heterogeneous data sources easier to use, several standard XML exchange formats for sharing interaction and pathway data have been developed. The Systems Biology Markup Language (SBML) [13] and CellML [14] represent mathematical models of pathways designed for quantitative simulation of concentration profiles of components over time. The Proteomics Standards Initiative's Molecular Interaction (PSI-MI) format enables exchange of molecular interaction data sets [11]. Finally, the Biological Pathway Exchange (BioPAX) format enables exchange of biological pathways in general [15].

As more pathway databases make their data available in standard formats, it is easier to aggregate pathway information from multiple sources. A convenient single point of access for pathway information would provide researchers with a more complete and powerful view of biological networks and cellular machinery. Database and software systems that work towards this goal include pathway databases with original content, such as those listed in Table 2 and in Pathguide [16]; network analysis software applications that support multiple import formats and local interaction data stores, like PIANA [17], Ondex [18] and Cytoscape [19] and biological network data integration systems, similar in scope to sequence integration systems like SRS [20], like Biozon [21], SigPath [22], Biomodels [23], Atlas [24] and PKB [25]. cPath is part of the latter class, but is the only system that is jointly open source, easy to locally install, contains identifier resolution services, provides web based front end and web service API and includes support for pathway and molecular interaction information available in both the PSI-MI and BioPAX standard database file formats.

**Table 2:** External databases successfully imported into cPath.

**Database Name**	**Database Type**	**URL**	**Release Date Tested**	**Data Format used for cPath Import**	**Database Stats**
**Cancer Cell Map**	Signaling Pathways		April, 2006	BioPAX, Level 2	Pathways: 10 Interactions: 2,104 Physical Entities: 899
**Database of Interacting Proteins (DIP) [54, 55]**	Protein-Protein Interactions		April, 2006	PSI-MI, Level 1	Interactions: 54,511 Physical Entities: 19,003
**Ecocyc [56]**	Metabolic and Signaling Pathways	Registration required to download data	March, 2006	BioPAX, Level 1	Pathways: 224 Interactions: 4,644 Physical Entities: 3,511
**Human Protein Reference Database (HPRD) [57]**	Protein-Protein Interactions	Registration required to download data	Sept, 2005	PSI-MI, Level 1	Interactions: 4,450 Physical Entities: 12,226
**IntAct [46]**	Protein-Protein Interactions		May, 2006	PSI-MI, Level 1	Interactions: 67,816 Physical Entities: 28,006
**KEGG [58, 59]**	Metabolic Pathways		July, 2006	BioPAX, Level 2	Human Data Only Pathways: 108 Interactions: 3,285 Physical Entities: 2,018
**Molecular Interaction Database (MINT) [45]**	Protein-Protein Interactions		May, 2005	PSI-MI, Level 1	Interactions: 44,904 Physical Entities: 16,325
**Reactome [47]**	Metabolic and Signaling Pathways		May, 2006	BioPAX, Level 2	Human Data Only Pathways: 775 Interactions: 2,962 Physical Entities: 3,197

## Implementation

### Technical Architecture

cPath is built using a traditional three-tier web architecture. The first tier consists of the open source MySQL database [26], and full-text index files generated by the open source Lucene index engine, which also handles most query tasks for the web service [27]. The second tier consists of a Java servlet application, which uses the Jakarta Struts Library [28] for cleanly separating application logic from HTML/XML presentation. The second tier also uses the Xerces Java XML parser [29], the open source JDOM library [30], and ARP: Another RDF Parser [31]. In-memory caching is provided via the open source Ehcache tool kit [32], and database pooling is provided by the Apache Database Connection Pool (DBCP) [33]. Real-time logging is provided by Apache Log4J [34], unit tests are written in JUnit [35], functional tests are written in Anteater [36], and the entire build process is fully automated via Ant [37] and Cruise Control [38]. Detailed information about the cPath architecture, including graphical diagrams and definitions of the table structure, can be found in the Architecture Guide PDF on the cPath developer web site [39].

### Data Model

The core cPath data model consists of just three elements: biological entities, links between biological entities, and links to external databases. For example, when storing a PSI-MI interaction, such as "p53 binds to TP73", cPath splits the PSI-MI record into three biological entity records: one for the interaction, one for p53 and one for TP73. For each record, cPath stores the complete PSI-MI XML content associated with that entity. cPath then stores two internal links, one from the interaction record to p53, and a second from the interaction record to TP73. Finally, cPath stores external link records defined in the PSI-MI record for each of the biological entities; for example, cPath will store a link from p53 to its UniProt identifier, as well as links from p53 to any matching Gene Ontology (GO) terms if those references are present in the PSI-MI file. External links are supplemented by any unification links available in the identifier mapping system.

By maintaining a simple data model, and storing XML within a relational database structure, cPath is able to store data in multiple XML formats, including PSI-MI and BioPAX and the cPath table structure need not be updated when these formats change. cPath does not define any new XML formats, but rather adopts the data models of PSI-MI and BioPAX. Thus cPath can store any biological information represented by these standard exchange formats, from protein sequence, to experimental description, to thermodynamics information for biochemical reactions. Not all information will be displayed via the web interface, but all will be available in the XML returned by the web service API. Additional tables within cPath are used to support the three core entity tables. For example, cPath maintains an identity and reference table for mapping between identifiers, an external database table for creating hyperlinks to other biological databases, an organism table for storing basic organism data, an XML cache table for storing pre-computed XML assembly documents to speed loading of commonly accessed pages, and a log table for storing fatal errors in production mode.

## Results

### Overview

**Figure 1:**
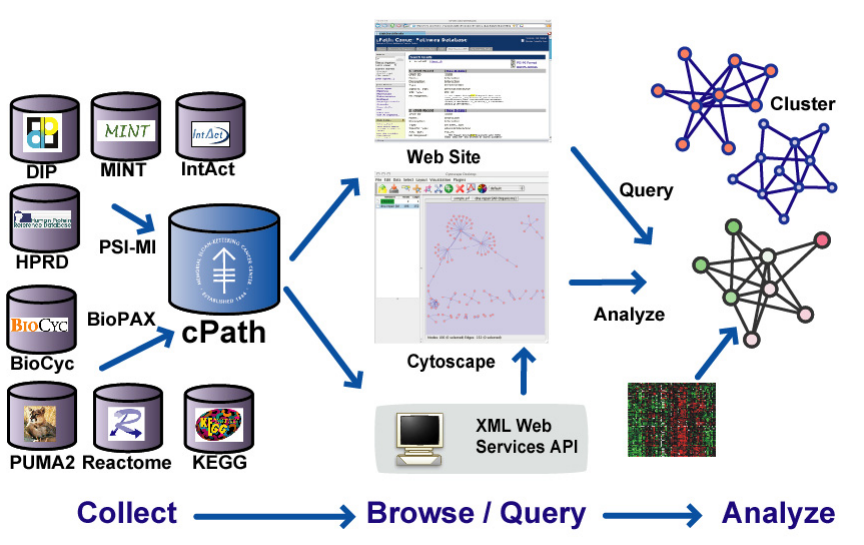
**cPath Open Source Pathway Database Software Overview**. cPath is open source software for collecting, storing, and querying biological pathways. At left, multiple databases can be imported into cPath via standard exchange formats. At right, cPath data can be viewed via a standard web browser or exported via an XML-based web service API, making cPath data available to third-party applications for pathway visualization and analysis.

Using cPath, researchers can import interaction and pathway data from multiple sources, access such data via a standard web interface, and export data to third-party applications via a standards-based web service (Figure 1). Biologists, computational biologists, and software developers can utilize cPath for content aggregation, query and analysis (see Table 1 for a list of target audiences). cPath can serve as a modular, core software layer in larger pathway information systems that are capable of visualizing, analyzing, and modeling biological pathways. All cPath software is freely available under an open source license for local installation and modification. The key features provided by cPath are detailed below.

**Table 1:** cPath is useful for biologists, computational biologists and software developers.

**Target Audience**	**cPath Usage**
**Biologists**	Browse and search for pathways of interest. Drill down from a pathway to member interactions and molecules. Search for a specific protein of interest, and look up its role in specific pathways. Use third-party tools to visualize and analyze pathways. For example, overlay expression data onto pathways of interest, and algorithmically identify regions of correlated activity.
**Computational Biologists**	Download all pathways in BioPAX format for global analysis using your own software. Install cPath locally to aggregate external and/or private data sets. Use cPath to distribute, annotate and share computationally predicted interaction data sets or pathway models.
**Software Developers/Tool Builders**	Build third-party software tools on top of the open cPath web service API, in order to programmatically access pathway data. For example, build software for visualizing pathways and organizing member molecules based on cellular location.

### Key Feature: Identifier Mapping System

A recurring problem in bioinformatics is linking related or identical data described by different databases when multiple database identifiers (primary keys) are used to refer to the same biological entity [40,41]. Interaction databases may use different identifiers for their proteins, RNA, DNA or small molecules (for example, a protein may be identified with a UniProt accession number, RefSeq accession number or an NCBI GI number). This use of multiple identifiers can significantly hinder the ability to seamlessly use data from diverse sources, such as to retrieve all interactions involving a protein from numerous data sources. Recognizing when protein records that use different database identifiers actually represent the same protein allows a query for the protein to correctly retrieve both original records [41]. To address this specific issue, cPath provides an identifier mapping system capable of storing equivalence between two or more identifiers. The system is pre-populated with identifier mappings loaded from external files. For example, a single protein unification mapping may map UniProt accession numbers to equivalent RefSeq accession numbers. Identifier mapping files are simple tab-delimited text files that must be loaded into cPath prior to import of any interaction or pathway data sets. With some scripting ability, cPath identifier mapping files can be created from external database resources, such as Alias Server [42], the EBI International Protein Index (IPI) [43], or Ensembl BioMart [44]. Sample protein unification files, derived from the IPI Protein Cross-References dataset, are available for download from the cPath web site. cPath also uses identifier equivalences available in imported pathway datasets that contain multiple database references for the same interactors.

Importantly, cPath also provides a similar service for storing relationships between non-equivalent, but related biological entities. For example, a researcher can import a UniProt to Affymetrix mapping file, then when a new protein with a matching UniProt identifier is subsequently imported into cPath, it is annotated with all known Affymetrix probe set identifiers. This is useful for tools that link gene expression data to pathways.

### Key Feature: Scalable Pathway Data Aggregation

To support data aggregation from multiple databases, such as to create custom integrated sets of pathways for local use, cPath supports the PSI-MI [11] and BioPAX [15] exchange formats. As more databases make their data available in either of these two standard formats, cPath becomes increasingly useful. As some popular pathway databases do not permit public redistribution of their data, it is difficult for central websites to collect a complete set of pathways for research use. A local installation of cPath is one way to effectively collect and access all of this data. For example, we have successfully aggregated data from MINT [45] and IntAct [46], resulting in a final data set of over 20,000 proteins, and more than 84,000 interactions. cPath has also been successfully used to store all human pathways from Reactome [47], and one of our active users has used cPath to successfully store over 1 million interaction records. A complete list of external databases, which have been successfully imported and stored using cPath is provided in Table 2.

cPath supports PSI-MI format Level 1 and BioPAX format Levels 1 and 2. Level 1 of PSI-MI represents protein-protein interactions. Level 1 of BioPAX represents metabolic pathways, Level 2 adds support for molecular interactions and post-translational protein modifications, such as those supported by PSI-MI, and future levels will add support for signaling pathways, gene regulatory networks and genetic interactions by the end of 2006.

### Key Feature: Standardized Web Interface for Browsing and Querying Pathways

**Figure 2:**
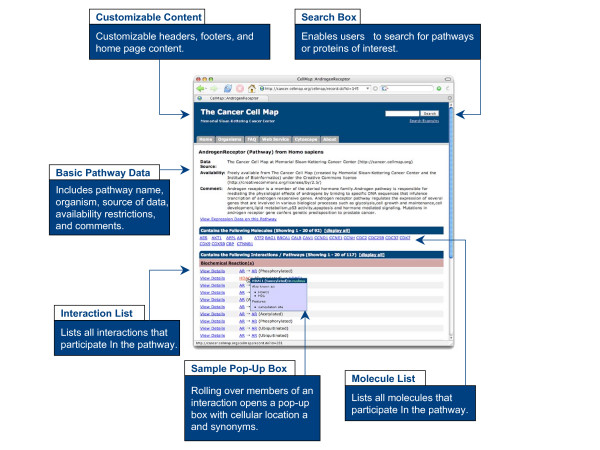
**Browsing Pathways using cPath**. cPath provides a web interface for querying biological pathways. This screenshot is of the human Androgen receptor pathway from the Cancer Cell Map [48].

Once pathway data is stored in cPath, it is available for browsing via a standard web browser. For example, the Cancer Cell Map [48] currently uses cPath software as the underlying database, and makes available a set of cancer-specific pathways curated by the Institute of Bioinformatics in collaboration with Memorial Sloan-Kettering Cancer Center. Users of cancer.cellmap.org or any other cPath-powered site, have multiple options for querying. A user can begin with a list of pathways, or search for a specific pathway of interest, and drill down to view embedded components, such as biochemical reactions, complexes and proteins (Figure 2). Alternatively, a user can enter a search string, such as a protein name or identifier, in the query box, and link from the resulting query results page to interactors, interactions or pathways. cPath includes a full-text search engine that automatically ranks records based on relevance of search results and supports a simple language to define more complex queries, such as Boolean combinations of words.

### Key Feature: Standardized Web Service Interface for Application Communication

**Figure 3:**
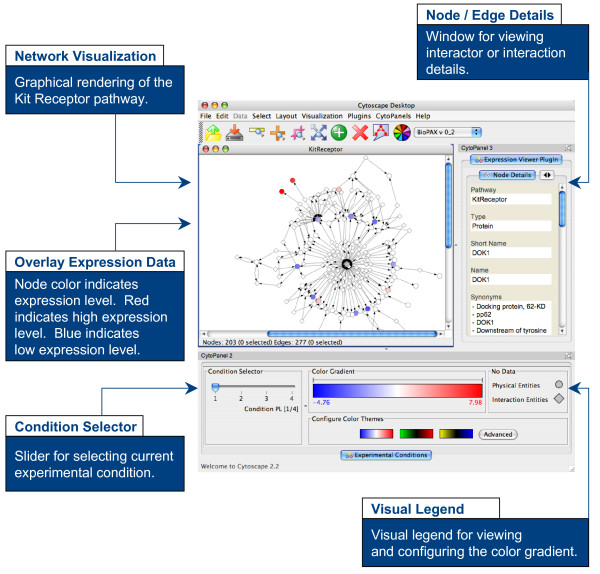
**Sample Cytoscape Plugin for Interfacing with cPath**. The Cytoscape Expression Viewer plugin enables researchers to visualize expression data on biological pathways. The plugin utilizes the cPath web service API to retrieve pathway data, such as the Kit receptor pathway from the Cancer Cell Map [48].

Data stored in cPath can be made available for query and export via a standards-based web service interface. For example, a third-party application can retrieve a list of all pathways stored in cPath, and then retrieve the full details of each pathway in subsequent calls back to cPath. The result of each query is a BioPAX or PSI-MI formatted XML data file that can be parsed and used by the application. By exposing all data via a standards-based web service interface, cPath enables interoperable communication with other software modules, and enables third-party applications to more easily build and expand tools for visualization, analysis and model simulation. For example, the cPath plugin for Cytoscape [19] enables researchers to download and visualize protein-protein interaction networks. A second Cytoscape plugin enables researchers to view gene expression data along a color gradient and in the context of known biological pathways retrieved from cPath (Figure 3). The cPath web service is not tied to a specific operating system or programming language, and uses a REST-based (Representational State Transfer) architecture [49], which has only two requirements: queries must be specified as Internet URLs, and response documents must be specified as XML documents. This REST-based approach is considerably simpler than other web service options, such as SOAP [50], and enables developers to interactively experiment with the cPath web service with just a standard web browser. This helps lower the development effort required to interface with cPath, while simultaneously maintaining platform and language independence.

### Key Feature: caBIG™ Interoperability

In addition to our own software engineering requirements, cPath meets specific interoperability and testing requirements of the National Cancer Institute (NCI) Cancer Biomedical Informatics Grid, or caBIG™. The goal of caBIG™ is to create a common infrastructure of interoperable tools and data specifically focused on cancer research [12,51], and software funded via caBIG is required to meet specific interoperability requirements. For example, silver-level compliance requires that the software use standard exchange formats, make all data available via well described APIs, and use standard messaging systems where appropriate [52]. Through caBIG™, cPath has been formally tested by a third-party partner institution, Oregon Health & Science University (OHSU). cPath was tested on multiple operating systems, and with multiple versions of the required software providing quality assurance (QA) of the entire software system. Since early 2004, the cPath web service has handled more than 537,000 queries from over 5,800 distinct hosts (IP addresses), mostly from Cytoscape users using the cPath query plugin.

### Key Feature: Open Source License, Local Installation and Customization

cPath is freely available under the GNU Lesser General Public License (LGPL) for academic and commercial use. cPath can be used to distribute pathway data on the Internet, or can be used to share private data locally within an individual lab, department or company. Stable releases of the cPath software are available for download, as are nightly snapshots of the latest code, which is not guaranteed to be stable, but may have new features compared to the last stable release. A complete administrator guide (available in PDF format) describes the step-by-step process for installing a new instance of cPath. Installation requires some computer system administration skill and ability to work with the command line, thus is geared towards computational biologists and software developers. Once installed, cPath can be administered via the command line using a Perl script wrapper around a Java administration program or via a web-based administration interface. After installation, typical set-up includes loading selecting identifier mapping and PSI-MI or BioPAX files and running the text indexer via the command line administration script or the web-based interface. Once these are done, cPath is fully functional and is ready for use. Any problems with import, such as errors in the input files, are detected by a validator and reported. The web-based administration provides several options for customizing the look and feel of cPath, including the ability to set global headers, footers, and home page content. Additionally, we maintain a public mailing list where new users can post questions related to the administration and use of cPath (cpath@googlegroups.com).

## Conclusion

cPath is a robust, scalable, modular, professional-grade software platform for collecting, storing, and querying biological pathways. It can serve as the core data handling component in information systems for pathway visualization, analysis and modeling. As cPath evolves, we hope to attract new collaborators and developers to contribute to its open source development. We also envision that cPath will provide the software infrastructure for a large-scale, international effort to pool pathways from multiple sources into common repositories that provide convenient integrated points of access for the scientific community.

Future cPath software development will focus on increasing levels of data aggregation and linking. Currently, cPath is capable of integrating data at the interactor (e.g. protein) level by simply recognizing identifiers referencing the same protein, but it is incapable of detecting duplicate interaction or pathway records, resolving conflicting information and detecting semantic inconsistencies between different sources, all of which are required for true integration. Furthermore, while cPath is capable of importing both PSI-MI and BioPAX data, the web interface and web service must be set to support one of these exchange formats at a time. To support both exchange formats simultaneously, we will focus on creating translators between PSI-MI and BioPAX, and expand the roster of available web services queries to support both formats. cPath will also support PSI-MI Level 2.5, which expands the format scope to include other interactors, such as small molecules, DNA, and RNA and will support BioPAX Level 3.0 and future levels as they become available. We also plan to integrate BioPAX and PSI-MI validators, currently under development by others, into the cPath import pipeline to verify that all incoming BioPAX and PSI-MI records conform to their respective format definitions and to community best practices.

With the availability of tools for converting SBML and CellML to BioPAX developed by BioModels.net [23], we will be able to import SBML and CellML data sets present in BioModels.net directly into cPath. We will also support pathway export in SBML and CellML. Furthermore, in order to expand the number of other resources cPath is capable of linking to, we plan to integrate the MIRIAM (Minimal Information Requested In the Annotation of Biochemical Models) URI set. This community-derived data set provides a list of stable URIs and URL patterns for cross-linking bioinformatics resources and is currently used in SBML and BioModels.net [53].

## Availability and Requirements

• Project Name: cPath

• Project Home Page: 

• Operating System(s): Platform independent; tested on Windows, Linux and Mac OS X

• Programming Languages: Java

• Other Requirements: MySQL 4.0 or higher; Apache Tomcat Server 4.1 or higher; Apache Ant 1.6 or higher, Perl 5.0 or higher. All required software is open source and freely available.

• License: Free for academic and commercial users under the GNU Lesser General Public License (LGPL).

• Example site running cPath software: Memorial Sloan-Kettering Cancer Cell Map, .

## Authors' contributions

EC managed the overall software development process for cPath, wrote most of the code for cPath, and wrote the first draft of this manuscript. GB conceived of the cPath project, managed the scientific and technical goals, and helped draft the manuscript. BG contributed to the web interface for displaying BioPAX records, built the administrative interface for customizing cPath web pages, and built the cPath plugin for overlaying expression data onto biological pathways. CS conceived of the cPath project, and provided overall scientific and technical guidance. All authors read and approved the final manuscript.
